# A Case Report and Literature Review to Aid in the Management of Trunnion Failure in Hip Arthroplasty Patients: Can Trunnionosis and Prosthetic Joint Infection Co-Exist?

**DOI:** 10.7759/cureus.5544

**Published:** 2019-08-31

**Authors:** Vivek Jagadale

**Affiliations:** 1 Orthopedics, University of Arkansas for Medical Sciences, Little Rock, USA

**Keywords:** trunnionosis, metallosis, prosthetic joint infection, bloody aspirate, arthroplasty, metal-on-polyethylene

## Abstract

Trunnionosis is a type of corrosion and wear at the head-neck taper junction of the femoral implant, and it can be a slow and silent catastrophe. Simultaneous prosthetic joint infection (PJI) is occasionally possible based on the fulfillment of a few of the minor criteria from the Musculoskeletal Infection Society (MSIS), but the existing literature lacks adequate evidence to support that the infection actually exists. We are presenting a case of an 82-year-old man with right total hip arthroplasty performed over a decade prior to presenting to the emergency room with a sudden-onset pop followed by groin pain and difficulty in walking. Radiographs showed a dissociated femoral implant at the level of trunnion with malalignment and heterotopic ossification. Metal Artifact Reduction Sequence MRI of the right hip showed mixed type-two and type-three pseudotumors, and atrophy of surrounding abductor muscles. The erythrocyte sedimentation rate was within normal limits, C-reactive protein was borderline raised, and serum cobalt-chromium levels were elevated without any signs of systemic metal toxicity. Hip joint aspirate revealed blood-stained fluid flooded with red blood cells, leukocytes and neutrophils, and a positive alpha-defensin assay. These findings were interpreted as positive for prosthetic joint infection. Intraoperatively, there was severe wear of the inferomedial aspect of the femoral head-neck junction and extensive metallosis throughout the right hip. Tissue and fluid specimens were sent for cultures, sensitivities, and histopathology for pseudotumor and infection evaluation. An articulating antibiotic spacer was then placed with the intent to perform a staged reconstruction of the femur and right acetabulum. Final synovial, bone, and soft tissue cultures, as well as histopathological photomicrograph of the tissue slides, were negative for infection. This case demonstrates the striking features of metallosis associated with trunnion failure of a metal-on-polyethylene total hip joint prosthesis that was simultaneously showing signs of prosthetic infection by satisfying the minor criteria according to the latest guidelines by the MSIS with a strikingly high cell count of red blood cells in the synovial fluid exam, indicating inflamed hyper-vascular pseudotumors vs. hemarthrosis vs. bloody tap. Diagnostic dilemma led by positive synovial fluid alpha defensin, high synovial neutrophil and white cell count results with negative final cultures or infection on histological slides raises concern that infection was not present and two-stage revision arthroplasty with six weeks of antibiotics was not necessary along with increased risk of morbidity, mortality as well as cost of care.

## Introduction

Corrosion and wear at the head-neck taper junction of the femoral implant in metal-on-polyethylene total hip arthroplasty (THA) with local release of cobalt, chrome, and titanium ions-though considered rare-can be a slow and silent catastrophe. The released metal particles can build up in soft tissue, leading to tumor-like reactions that leak into the bloodstream, which can ultimately lead to metal poisoning that manifests as depression, suicidal tendencies, and psychosis [1].

Considerable controversy exists regarding the evaluation, radiological interpretation, and therapeutic approach in patients who present with vague symptoms and have undergone such a prosthesis placement. The initial evaluation includes a clinical assessment, a blood metal-ion level check, a synovial fluid exam, and imaging. However, levels of metal ions in the blood do not correlate with local adverse reactions visualized on MRI or during surgery, and a large portion of pseudotumors may be asymptomatic near operated hip but symptomatic from metal poisoning and vice versa [1,2]. These factors emphasize the importance of careful analysis to identify clues for implant failure, which could impact the surgeon’s decision making, surgical outcome, patient recovery, and satisfaction [2,3].

## Case presentation

**Figure 1:**
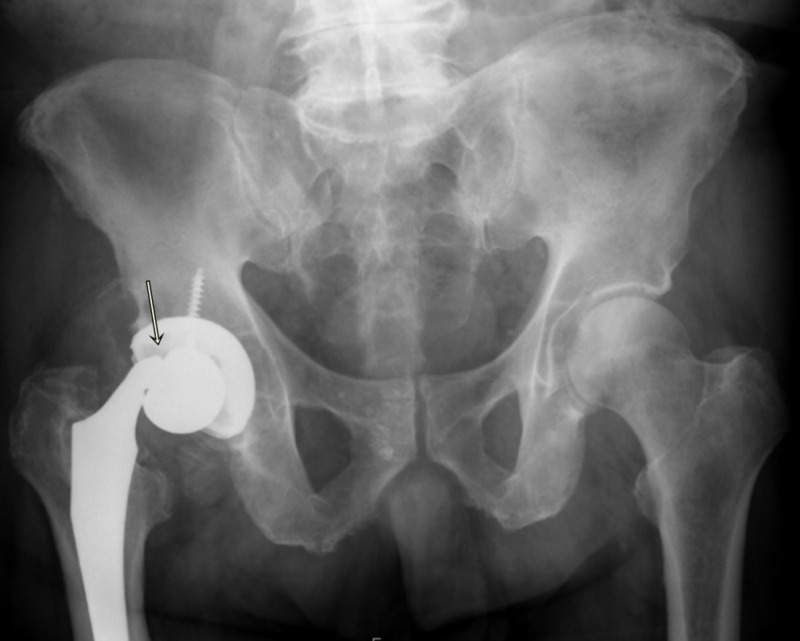
Anteroposterior radiograph of the pelvis with head neck dissociation of the right hip implant

**Figure 2:**
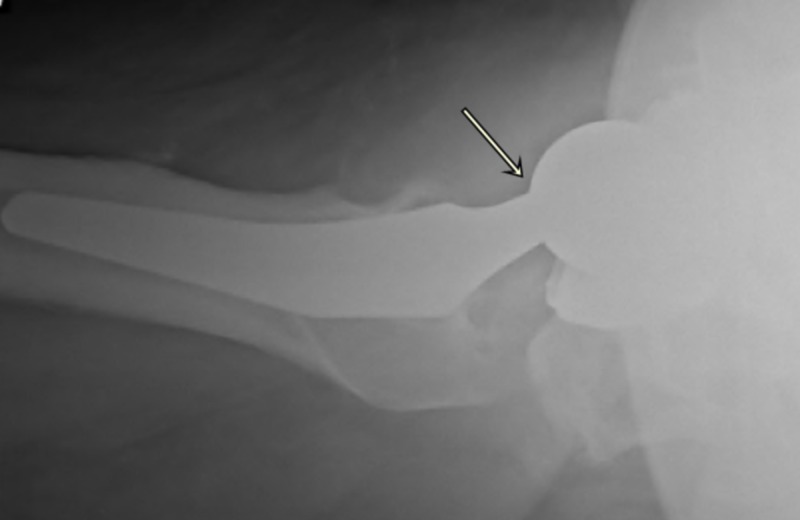
Lateral radiograph of the pelvis with head neck dissociation of the right hip implant

An 82-year-old obese (BMI: 41 kg/m^2^) man, with a past medical history of hypertension, hypothyroidism, osteoarthritis, and interstitial lung disease, with a history of right THA, performed 10 years ago (Stryker Hemispherical cluster hole shell, 60 mm, Accolade-II, size five hip stem paired with +2V40 40 mm Cobalt-Chrome head), had been experiencing a intermittent groin and lateral trochanter pain sensation in his hip, on and off, since initial surgery. He suddenly experienced a ‘pop’ in his right hip while getting up from the bed, followed by difficulty in standing and walking. Following this episode, he began experiencing moderate to severe pain in the right hip groin and was able to bear only minimal weight on that joint with the support of a walker, so he decided to go to the regional hospital emergency room. On examination, he was found to have normal-looking skin around the hip with a nicely healed old surgical scar from the anterolateral approach. No swelling was noticed around the hip joint, there was no open wound, there were no obvious signs of infection, and the distal neurovascular status was found to be completely intact. On further examination, he had a 2-cm true shortening on the right leg, painful range of motion of the hip from 70-degree flexion, zero-degree extension, 20 degrees abduction and adduction each, internal rotation of 10 degrees and external rotation of 30 degrees. On initial right hip radiographs, there was a dissociated femoral head implant at the level of trunnion with malalignment and shortening with no obvious signs of peri-prosthetic osteolysis, suggestive of infection or component loosening (Figures 1, 2).

On routine lab investigations, there was low hemoglobin (9.1 g/dL), elevated white blood cell (11,000 /µL), erythrocyte sedimentation rate (14 mm/h), and C-reactive protein (CRP; 11 mg/L) levels. His serum chromium level was 2.3 µg/L (reference range, < 5.0 µg/L), and serum cobalt level was 5.9 µg/L (reference range, <1.0 µg/L). The patient displayed no systemic signs of cobalt toxicity or infection, but, given his high CRP levels, we decided to proceed with hip aspiration. Right hip joint aspirate revealed dark, turbid fluid with elevated neutrophils (98%), red blood cells (10,000/µL), and leukocytes (11,100/µL). A Synovasure® panel (Zimmer Biomet, Claymont, DE) was positive for alpha-defensin.

**Figure 3:**
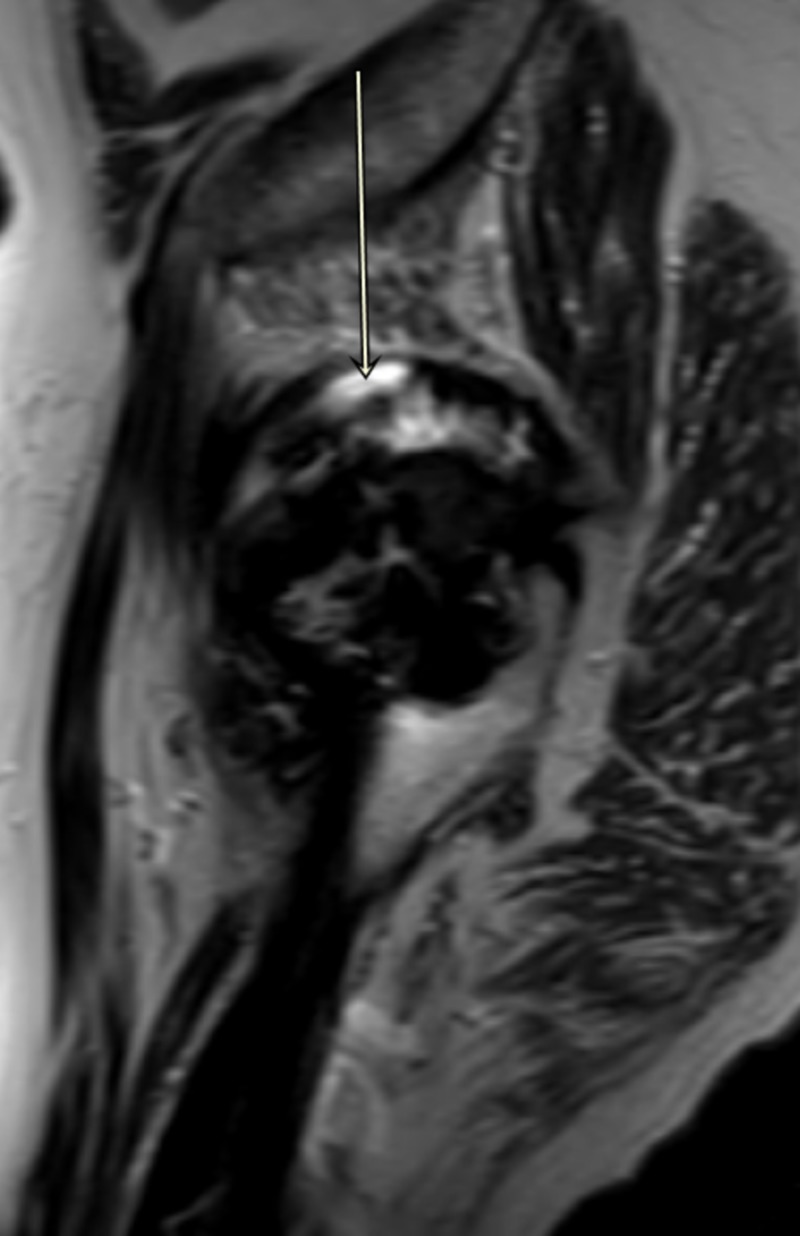
MARS image of right hip showing pseudotumor (Sagittal T2) MARS, magnetic artifact resonance sequence

**Figure 4:**
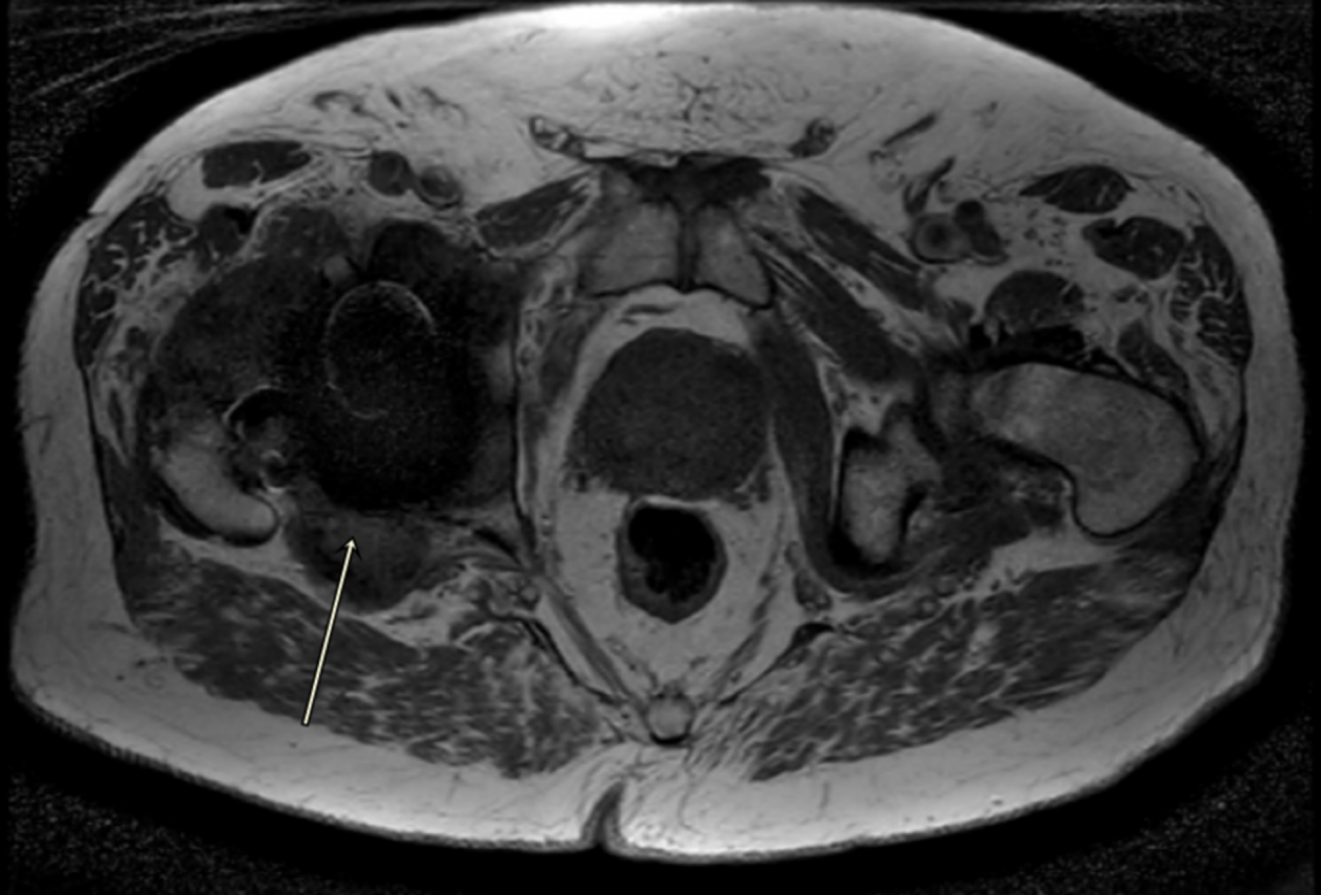
MARS image of right hip showing pseudotumor (Axial T2) MARS, magnetic artifact resonance sequence

**Figure 5:**
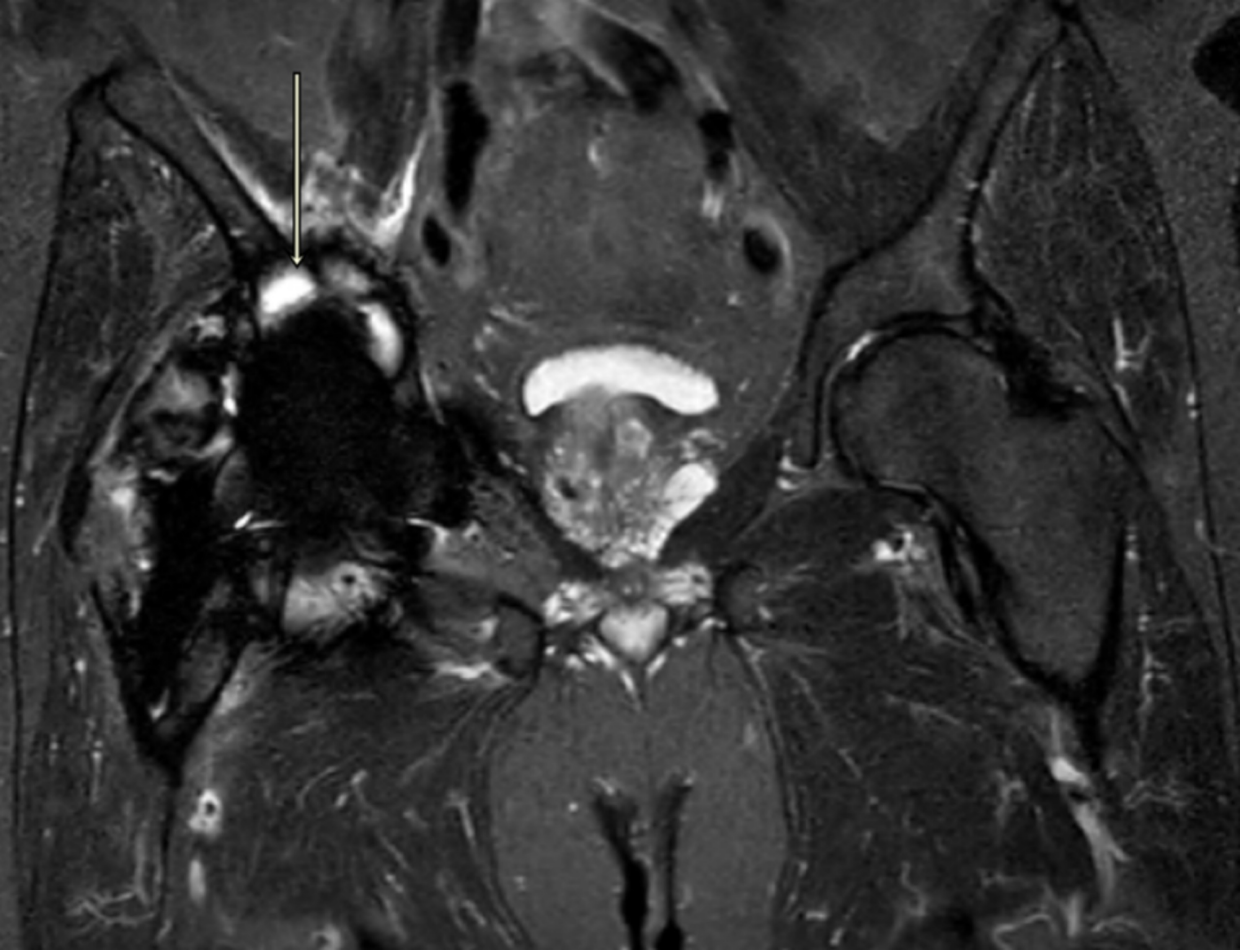
MARS image of right hip showing pseudotumor (Coronal T2) MARS, magnetic artifact resonance sequence

Magnetic artifact resonance imaging of the right hip showed large complex, mixed type-two and -three pseudotumors around the joint with atrophy of adjacent muscles, gluteus minimus, obturator internus and externus, and piriformis muscle (Figures 3-5). 

**Figure 6:**
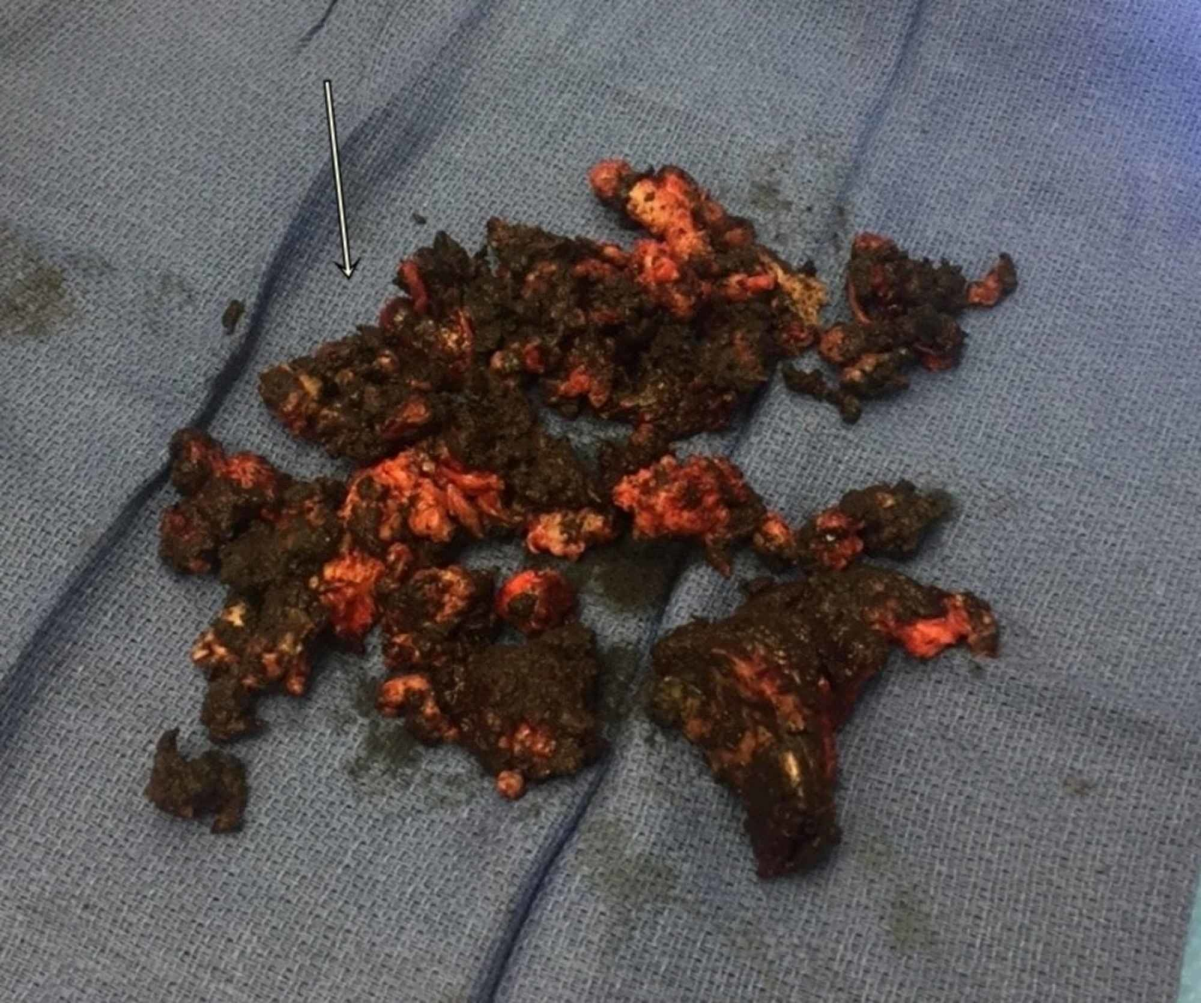
Intraoperative photograph of head-neck taper failure debris from metallosis

**Figure 7:**
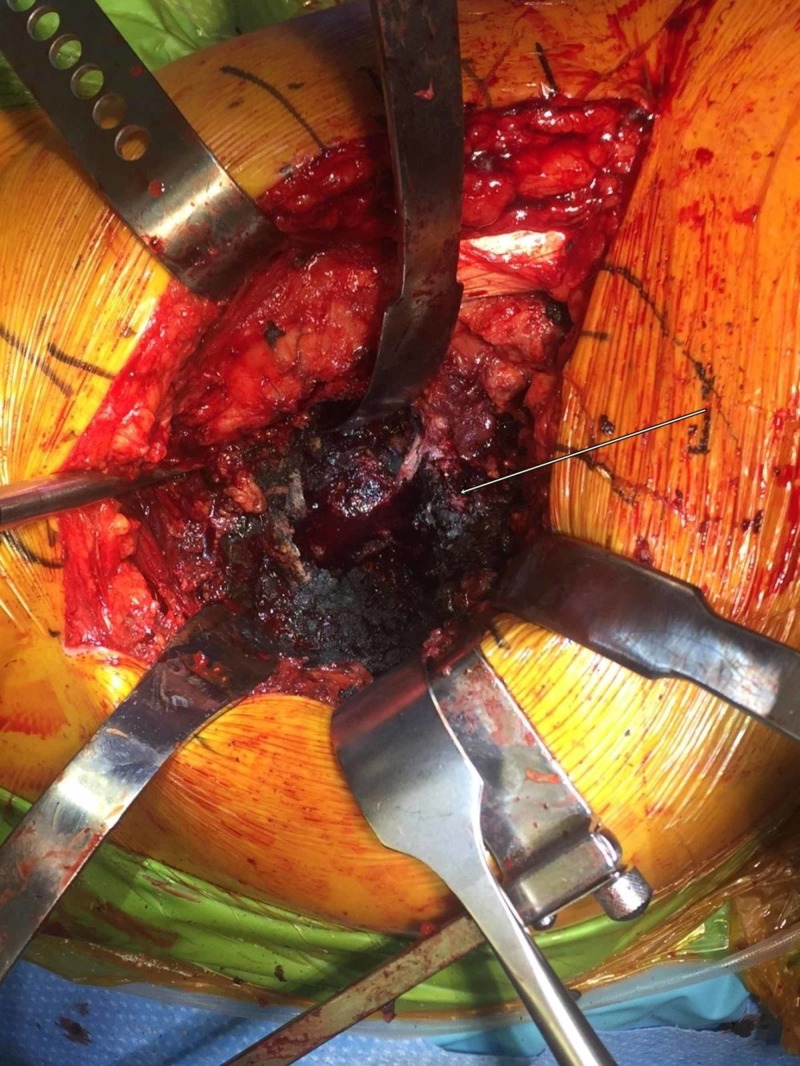
Intraoperative photograph of gross examination of tissue staining of pseudotumor from trunnion failure

Based on the presentation, the differentials were metallosis/trunnionosis, prosthetic joint infection (PJI), loosening of the implant with a periprosthetic fracture, or aseptic loosening from non-metal particle disease. The blood, aspirate, and imaging results were discussed with the patient; after our discussion about treatment options, their benefits, risks, alternatives, and complications, the patient consented for a revision hip surgery. The procedure was done with a posterior approach; no obvious purulence in the hip joint was identified, but dark, turbid hip joint fluid fountained out of the joint upon arthrotomy. Intraoperative findings were obvious for dissociation of the femoral head from the neck taper with the wear of trunnion with no implant fracture. Severe metal debris and tissue staining with necrosis from metal-induced adverse local tissue reaction (ALTR) and pseudo capsule formation were noted throughout the hip (Figures 6, 7).

**Figure 8:**
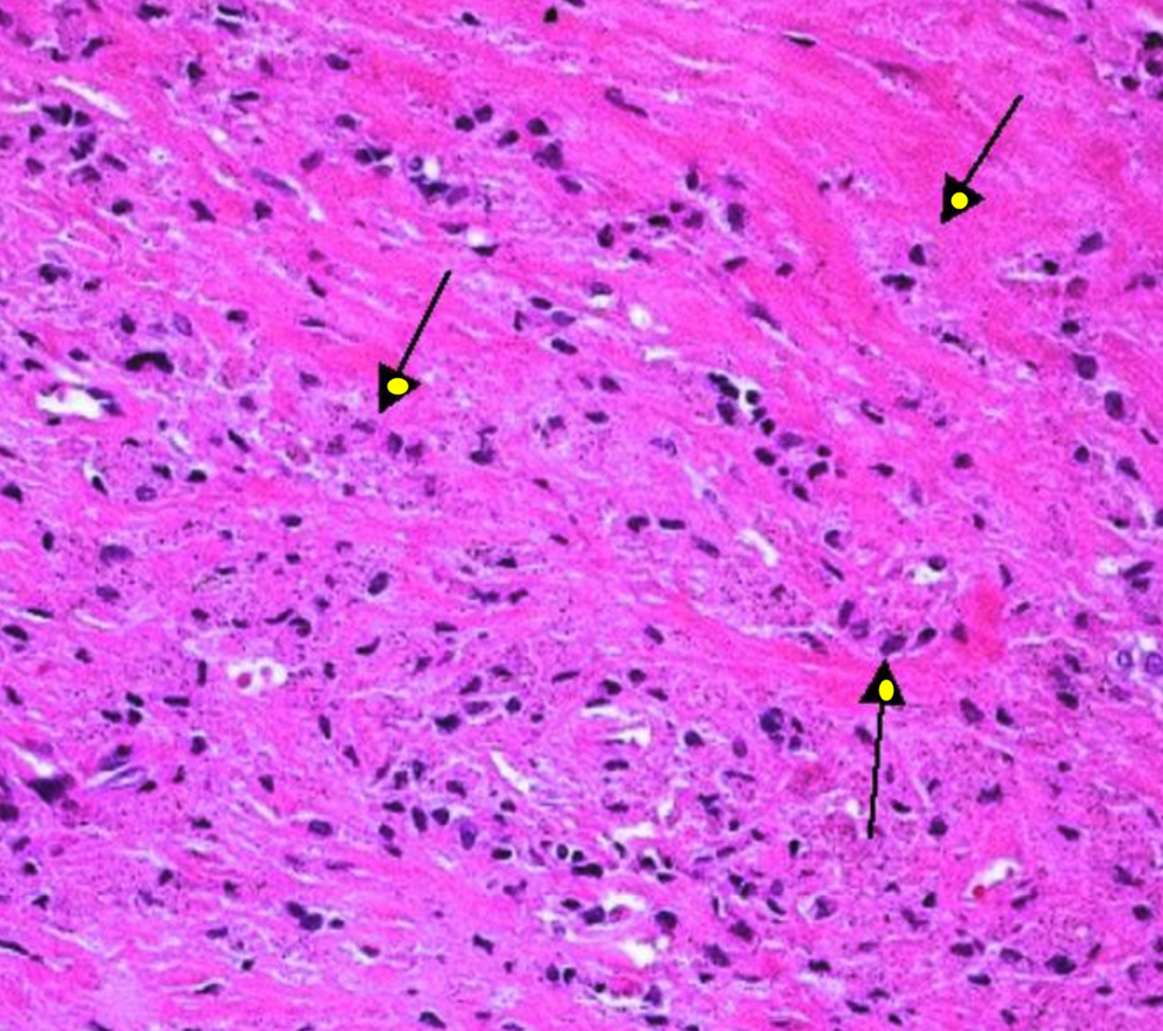
Photomicrograph showing Type 1 histology with dark pigmented foreign body deposits in fibroconnective tissue with necrosis consistent with metal particle disease and without signs of infection Hematoxylin and Eosin Stain, original magnification 100x

The acetabular component was removed with minimal bone loss; a superficial layer of bone was reamed out and curetted, necrotic tissue was removed, and the cavity was thoroughly irrigated. The femoral stem was removed easily with good proximal femoral bone stock remaining. Thirty percent abductor muscle damage due to severe metallosis was noted. Extensive sharp excision and curettage were used to remove a large amount of necrotic soft tissue, and gross photographs were obtained. Multiple fluids, soft tissue, and bone specimens were sent for cultures and histopathological evaluation for particle disease, ALTR, pseudotumor, and infection. Intraoperative specimens showed dense fibrotic tissue with scattered pigmentation and associated necrosis without evidence of an increase in inflammatory cells, abscesses, or vasculitis (Figure 8).

After thorough debridement and bone bed preparation, an articulating antibiotic cement spacer was placed with the intent to perform a staged reconstruction of the femur and right acetabulum later when the infection resolves. Stryker-cemented hip implants including all polyethylene-constrained acetabular liner size 58, size three cemented hip stem, 28 mm Cobalt chrome head, three packs of Palacos bone cement (Heraeus Medical, LLC; Yardley, PA) with 4 gm vancomycin, along with 2.6 gm tobramycin per pack were implanted using the non-pressurized cementing technique. Copious irrigation with pulsed lavage for a total of 9 L of 0.9% plain normal saline irrigation was done before and after the spacer placement. The wound was closed in layers in a regular fashion, and adequate hemostasis was achieved, so the drain was not necessary. Postoperative radiographs showed good alignment between the femoral and acetabular components. The patient tolerated the procedure well and was subsequently admitted to the hospital for pain control, routine antibiotics, and physical therapy.

A 325 mg once-daily aspirin tablet was started postoperatively as prophylaxis for deep vein thrombosis. The patient was ambulatory 24 hours after surgery and worked on joint weight bearing as tolerated at the direction of a physical therapist. Based on suspicion of simultaneous PJI on the grounds of partially fulfilling the minor criteria of the Musculoskeletal Infection Society (MSIS) definition, an infectious disease attending was consulted, and the patient was started on vancomycin (trough level 15-20) and ceftriaxone 2 gm per 24 hours for six weeks. A peripheral intravenous (IV) catheter line was placed so that the patient could be discharged and continue his IV antibiotic regimen. Final cultures were negative with fungal and acid-fast bacilli (AFB) cultures still pending, and the histopathological examination was showing zero to two polymorphs per high power field (HPF) in six different specimen fields. The patient was discharged home on the fourth postoperative day with continued IV antibiotics and deep vein thrombosis prophylaxis regimen. The patient had a good range of motion in the right lower extremity with intact sensations. He was instructed to continue physiotherapy at home. On follow-up evaluation at two, six, and 12 weeks postoperatively, the patient was doing well. Final cultures were negative for bacteria, acid-fast bacilli, and fungi. However, an aggressive antibiotic regimen was continued to treat peri-PJI, assuming it was a culture-negative infection. The patient was allowed ambulation with a walker and could fully bear weight on the right lower extremity immediately after surgery. The patient was discharged home on postoperative day four and subsequently monitored via follow-up at two, six, and 10 weeks in the clinic and was progressively doing well. At 15 weeks after stage one surgery, he underwent stage two revision surgery. Unfortunately, on postoperative day two, he sustained a massive pulmonary embolism, required placement on a ventilator and intensive care monitoring with two more months to recover. On the last follow-up at one year, the patient was ambulating half a mile daily using his cane with mild Trendelenburg gait from his abductor weakness, his incision was nicely healed, and his hip pain had significantly improved. The patient was made aware of the findings, and his permission was obtained for submission of this case report.

## Discussion

Head-neck junction failure in hip arthroplasty is defined as wear of the tapered femoral head and/or stem neck in femoral prostheses, leading to a gradual loosening and dissociation of components with neighboring tissue reaction that could mimic infection or tumor, although they rarely coexist [2,4,5]. Ongoing corrosion and wear at the head-neck junction can cause shedding of metal particles into the joint, leading to a condition called metallosis, which is defined by clouded or stained joint aspirate or visible dark staining of soft tissues on gross examination [5,6]. These metal particles are cytotoxic when phagocytized, leading to soft tissue and bone necrosis. The sequelae of this phenomenon have been defined as an aseptic lymphocyte-dominant vasculitis-associated lesion, also known as a pseudotumor, radiologically appear as a walled-off fluid collection, and clinically manifest in joint distention and vague pain [5]. Patients should be monitored for cobalt or chromium toxicity, which may present as worsening depression, psychosis, lack of sleep, appetite, concentration, and memory and weight loss [2].

Modern THA is a very successful operation with 96% overall patient satisfaction; however, implant designs, component choices, component preparation, sizing, and abnormal placement in the body may have a detrimental effect on the longevity of the implant and can lead to eventual early failure [5]. Modular hip prostheses with interchangeable femoral heads and stems have replaced monobloc implants in recent years, allowing orthopedic surgeons to modify implant length and offset in order to avoid hip instability and abductor dysfunction. However, these modular head-neck junctions have been shown to exacerbate torque and wear at the tapered trunnion joint, increasing the risk of failure. Larger diameter femoral heads in total hip replacements are known to increase stability by way of decreasing femoral head subluxation. However, the use of larger femoral head prosthetics increases stress on the trunnion taper, which has been correlated with an increased incidence of trunnion failure [2,3,6].

Patients with metal-on-metal pseudotumors present primarily with pain, and thus a wide differential for periprosthetic joint pain must be considered [7-9]. The important differential diagnoses for the typical MRI findings include PJI and abductor tendon avulsion-associated fluid collection. Septic joints and tendon avulsion-associated fluid collections are typically not as well defined and lack the low signal intensity rim that is typical of pseudotumors [1,10]. Importantly, tendon avulsion-associated fluid collections and pseudotumors may coexist in postoperative patients. Other differentials include prosthetic subsidence and aseptic loosening, periprosthetic fracture, insufficiency fracture, and local neoplastic processes. Joint aspiration was performed in this case to rule out PJI, and serum chromium and cobalt levels were used to confirm metallosis [5].

A treatment algorithm indicating joint revision in cases of trunnionosis with radiographic abnormalities and elevated serum chromium/cobalt levels has been suggested, but confusion still exists due to the complexity of the case presentation [3,5]. Extensive debridement of affected tissues must be undertaken. In cases where trunnion failure is known or encountered during surgery, the femoral stem may be retained with the addition of titanium sleeve to the trunnion if there is no evidence of severe corrosion, due to the morbidity associated with removal of well-fixed stems [9,11]. Cobalt-containing femoral heads are also exchanged for ceramic- and titanium-containing components.

PJI can be a devastating complication of hip arthroplasty, and at times, the diagnosis could be very elusive [12]. The major and minor diagnostic criteria set by MSIS have been very helpful in most situations; however, diagnosis could get tricky in simultaneous particle disease-forming hyper-vascular pseudotumors, hemarthrosis, bloody aspirate, or low-grade infections from fungus or low-virulence bacteria, which could have a significant impact on surgical planning, complications, morbidity, mortality, and cost of care [10]. Alpha-defensins are antimicrobial peptides released by neutrophils in response to pathogens, and they have been used more often than ever to identify such infections [13,14]. They can be measured in synovial fluid, lungs, intestines, or blood and are released from dying neutrophils in response to a bacteria, virus, fungi, or foreign bodies. Alpha-defensin use as a PJI diagnostic marker was introduced first by Deirmengian et al. in 2014 [15]. Aspirates from prosthetic joints with metallosis demonstrate approximately a 30% false-positive alpha-defensin rate [12]. A bloody tap can skew the synovial fluid neutrophil as well as leucocyte count, increasing the possibility of false-positive tests that could result in a false interpretation of simultaneous PJI and the absence of other signs of infection and subsequent ‘unnecessary’ extensive two-stage surgeries.

Trunnionosis from head-neck taper corrosion and failure with a simultaneous PJI that conclusively satisfies major MSIS criteria have not been reported in the literature. Therefore, when the serum and synovial fluid markers are abnormal in the setting of metallosis, PJI needs careful ruling out or consideration of the minor diagnostic criteria, especially in the setting of bloody aspirate (i.e., excess red blood cells in synovial fluid analysis). This could abnormally raise synovial leukocytes as well as polymorph count and would have a significant impact on the surgery planning, implant choices, complications, morbidity, and recovery, as well as the patient’s return to activities of daily living and long-term satisfaction, along with implant survival.

Moraweitz et al. classify the histological findings of the peri-prosthetic membrane into four different types [16]. Type I histology is characterized by the presence of foreign-body particles, macrophages, multinucleated giant cells, typical for wear-particle disease. Type II histology is defined by the presence of neutrophils and a very few foreign body particles typical for infectious etiology. Type III is a combination of Type I and II, while Type IV histology is indeterminate. This system highlights the fact that the inflammatory response to infection may be present in conjunction with other histological findings and must be carefully ruled out in multiple HPFs. Grammatopoulos et al. noted a good correlation between microbiology and histology with regard to the diagnosis of infection [17]. All cases of microbiologically confirmed PJI had a heavy neutrophil polymorph (NP) infiltrate (>5 NPs per HPF) in periprosthetic tissues. They noted occasional NPs in some cases of aseptic failure, mostly <1 NP per HPF on average, but, in one case, up to four NPs per HPF was noted.

They have advised caution in interpreting the number of polymorphs to diagnose PJI in the context of a failed metal-on-metal hip arthroplasty and suggested that the histologic findings should always be correlated with microbiology results in the evaluation of all cases of metal-on-metal hip revision arthroplasty.

In this particular case, the patient did not exhibit systemic signs of infection or metal toxicity; blood inflammatory markers were inconclusive for infection, serum cobalt and chromium levels were elevated suggesting metal particle disease, synovial fluid was flooded with red blood cells, leukocytes, neutrophils, and the synovial alpha-defensin immunoassay tested positive. These findings based on the MSIS minor criteria scoring were interpreted as positive for PJI; however, the final joint aspirate performed preoperatively, as well as six tissue cultures of intraoperative specimens from different locations and the histopathological examination of tissues did not find any abscess pockets or adequate polymorphs, thus ruling out an infection. We, therefore, foresee the need for more case-control studies to further reconsider the connection between minor criteria of PJI as established by MSIS in the setting of particle disease around joints and histopathological examination of intraoperative specimens to rule in or rule out the possibility of simultaneous PJI in the setting of metallosis, as most of these markers could be elevated due to medical comorbidities, hypervascular pseudotumors, bloody aspirates, and false-positive alpha-defensin assay [18].

## Conclusions

Trunnionosis with simultaneous PJI and presence of sinus tract communicating with the prosthesis and/or simultaneous positive tissue cultures is a rare phenomenon. Therefore, heightened caution must be used while interpreting the test results using minor MSIS criteria in favor of PJI, especially in the absence of obvious clinical signs favoring infection such as fever or sepsis. Otherwise, there may be a significant impact on morbidity, mortality, treatment outcomes, and cost of care. A major two-stage surgery could be avoided if clinico-radiological, laboratory, and histopathological parameters do not tee up to establish the diagnosis of concurrent infection.
